# nhanesA: achieving transparency and reproducibility in NHANES research

**DOI:** 10.1093/database/baae028

**Published:** 2024-04-15

**Authors:** Laha Ale, Robert Gentleman, Teresa Filshtein Sonmez, Deepayan Sarkar, Christopher Endres

**Affiliations:** School of Computing and Artificial Intelligence, Southwest Jiaotong University, No. 999, Xian Rd, Pidu Dist., Chengdu, Sichuan 611756, China; Center for Computational Biomedicine, Harvard Medical School, 25 Shattuck St, Boston, MA 02115, USA; Research Department, 23 and Me, Inc., 223 N Mathilda Ave, Sunnyvale, CA 94086, USA; Theoretical Statistics and Mathematics Unit, Indian Statistical Institute, 7 SJSS Marg, New Delhi 110016, India; The Promenade Dance Studio, Inc., 2605 Lord Baltimore Drive, Windsor Mill, MD 21244, USA

## Abstract

The National Health and Nutrition Examination Survey provides comprehensive data on demographics, sociology, health and nutrition. Conducted in 2-year cycles since 1999, most of its data are publicly accessible, making it pivotal for research areas like studying social determinants of health or tracking trends in health metrics such as obesity or diabetes. Assembling the data and analyzing it presents a number of technical and analytic challenges. This paper introduces the nhanesA R package, which is designed to assist researchers in data retrieval and analysis and to enable the sharing and extension of prior research efforts. We believe that fostering community-driven activity in data reproducibility and sharing of analytic methods will greatly benefit the scientific community and propel scientific advancements.

**Database URL**: https://github.com/cjendres1/nhanes

## Introduction

The National Health and Nutrition Examination Survey (NHANES) (1) is a pivotal program dedicated to assessing the health and nutritional status of both adults and children in the USA. Its value stems from its comprehensive approach that merges detailed interviews and thorough physical examinations. NHANES is administered by the National Center for Health Statistics, an integral part of the Centers for Disease Control and Prevention (CDC), tasked with generating crucial health and vital statistics for the entire nation.

**Figure 1.**
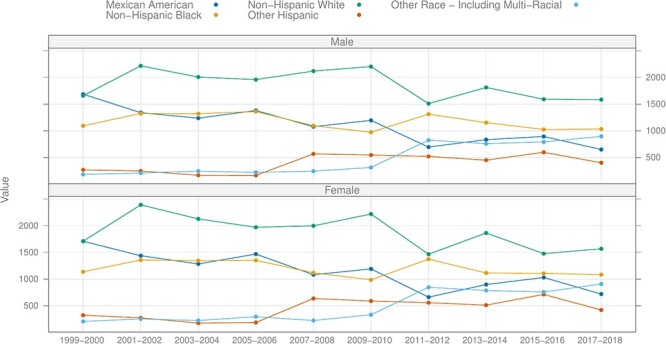
Number of participants by recorded ethnicity and gender by cycle, excluding the pre-pandemic (2017–March 2020) cycle.

Since 1999, NHANES has transitioned to a continuous survey format, distinctively termed ‘continuous NHANES’ to differentiate it from its preceding versions. Continuous NHANES surveys are grouped in 2-year ‘cycles’, with the inaugural cycle rolled out in 1999–2000. A PubMed (2) search reveals that NHANES is referenced almost 5000 times annually, highlighting its significance in the research community. The vast majority of the NHANES data are available for download from the CDC website, which also offers comprehensive guidance on data utilization, downloading procedures and analytic methodologies. Across cycle years (1999–2000 to 2017–2018), approximately 5000 participants joined the program annually, with data of 15 560 participants reported for the period 2017–March 2020, where activities were altered due to the coronavirus disease pandemic. Figure 1 shows the breakup of the number of participants by recorded ethnicity and gender, up to the 2017–2019 cycle.

NHANES employs a distinct sampling strategy that captures data from demographic groups often missing or underrepresented in many epidemiology studies. The sampling design intentionally oversamples demographic groups such as African Americans and Hispanic populations and age groups such as adolescents and the elderly. The sampling design is a stratified design, with 15 counties across the USA chosen as primary sampling units (PSUs) each year. Because of this design, analyses of the data should rely on proper use of appropriate survey sampling methodology (3) when making population-level estimates.

NHANES serves as a useful tool for studying both the prevalence of, and temporal shifts in, critical public health issues such as obesity. While each cycle is cross-sectional, one can examine the sequential order of cycles to get a sense of evolving population characteristics over time. The survey is not longitudinal as different sampling units are chosen for each cycle, so observed trends need to be computed and interpreted with some care.

This paper introduces the nhanesA R package, which facilitates the analysis of NHANES data by providing tools to search, download, extract and process data available on CDC’s NHANES website. It has proven to be of great benefit to investigators analyzing NHANES data (4–12). We show some elementary examples, illustrating the use of the package in the next section. The package also includes more detailed vignettes, describing in particular the appropriate use of survey weights and directing readers to an extensive array of online resources.

Replication of published papers remains a demanding endeavor. Even with well-intentioned authors, recreating tables and graphs from their papers may prove challenging. This difficulty often stems from a lack of specificity in reporting the extent and manner of data cleaning, the details of inclusion criteria, and specific phenotypic definitions. Furthermore, accurately detailing the extent to which the data were transformed or filtered during the analysis is difficult. While accurate textual descriptions of these processes can become unwieldy, they can be succinctly and unambiguously described through software. We have incorporated tools in the nhanesA R package to make it easier, in conjunction with tools such as Sweave (13), Rmarkdown (14, 15) and Quarto (16), to synchronize the software descriptions of the analysis with the outputs and to easily share the software with interested readers. We also provide a brief description, in the form of a supplement, of ways in which authors can enhance the reproducibility of their work by readers.

## Materials and Methods

### Data

The publicly available continuous NHANES data consists of over 1500 different tables or questionnaires. Each cycle surveys a distinct set of individuals using a cluster sample approach (17). Each cycle produces data tables in five categories: demographics, dietary, examination, laboratory, and questionnaire. There are also limited access data that are not publicly available and require a formal request for access.

The available data can be downloaded using Hypertext Transfer Protocol Secure requests from the CDC website. For each table, there are two components, the raw data which is provided in SAS (Statistical Analysis Software), XPT (Transport File) format (18) and a documentation file, in HyperText Markup Language (HTML), which describes the data variables and format. During the COVID pandemic, the CDC modified some procedures as documented in a study by Paulose-Ram *et al*. (19)

We next describe how the nhanesA R package offers effective and reproducible solutions to computational and analytical problems arising in epidemiological use of NHANES data.

### Search relevant variables and data files for analysis

While the CDC website provides search capabilities, we believe that using dedicated R-based tools offers analysts a way to programmatically prepare analyses and organize the research results. Within the nhanesA R package, we have incorporated advanced search utilities. Functions such as nhanesSearch(), nhanesSearchTableNames() and nhanesSearchVarName() have been crafted to streamline and optimize these search processes.

### Downloading data to your local machine

**Figure 2.**
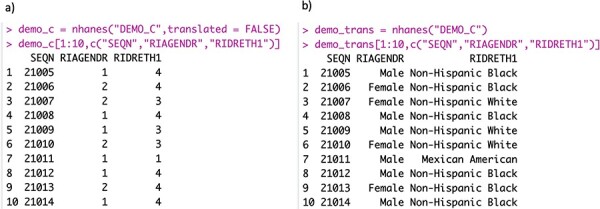
Panel (a) shows the raw data, where both gender and ethnicity are encoded as integers. Panel (b) shows the translated data.

Using the nhanesA R package, data can be downloaded directly from CDC servers into data frames, readying it for subsequent analysis. The function nhanes() takes the name of the table that is wanted and downloads it. Categorical variables, both ordered and unordered, are typically encoded as integers in the raw data available as XPT files, with the interpretations of these integer codes available in the accompanying HTML documentation files. By default, the nhanes() function ‘translates’ these integer codes into more easily interpretable text strings. In Figure 2, we show the translated and untranslated variables RIAGENDR and RIDRETH1 from the DEMO_C table, where, for example, the untranslated values for RIAGENDR coded as 1 and 2 are translated to male and female.

Apart from ease of interpretation, this translation is also important because if an untranslated categorical variable is used in a regression model then the variable would be treated as a continuous variable and the corresponding estimates would, in general, not be appropriate. The default translation in the nhanes() function can be suppressed by setting the argument translated to FALSE.

The nhanesA package includes several other utility functions. The functions available in version 1.0 of the package are briefly summarized in Table 1.

**Table 1.** List of functions in the nhanesA R package (version 1.0)

Functions	Descriptions
browseNHANES	Open a browser to NHANES
nhanes	Download an NHANES table and return as a data frame
nhanesAttr	Returns the attributes of an NHANES data table
nhanesCodebook	Display codebook for selected variable
nhanesCodebookFromURL	Download and parse an NHANES doc file from URL
nhanesDXA	Import DXA data
nhanesFromURL	Download an NHANES table from URL
nhanesManifest	Download and parse NHANES manifests
nhanesOptions	Options for the nhanesA package
nhanesSearch	Perform a search over the comprehensive NHANES variable list
nhanesSearchTableNames	Search for matching table names
nhanesSearchVarName	Search for tables that contain a specified variable

### Align tables within a cycle or across cycles

After downloading, the resulting data frames may be processed further using standard R data manipulation tools, including those from popular contributed packages such as dplyr (20), data.table (21), etc. For example, data from different tables within a cycle can be synchronized using the R function merge() with SEQN as the key. Additionally, one can align tables across cycles as long as the relevant data were collected in all the cycles. However, it is important to be cautious when merging or combining data across cycles as the names of the variables are not guaranteed to remain constant, and the actual questions may change over time. The CDC uses some naming conventions, but these are not always applied consistently.

### Use the survey weights to obtain valid estimates

NHANES employs a sophisticated four-stage sampling design, and proper analysis typically requires survey analysis methods that incorporate specific weights. These weights are crucial as they adjust for the particular sampling approach used in the data collection process. Each sample person is assigned a sample weight, reflecting the number of people in the broader population that the individual represents. To obtain valid estimates from the data, it is essential to apply these survey weights during analysis. By doing so, researchers account for the complex survey design and some potential biases, ensuring that the results are reflective of the entire population and not just the sampled individuals. There is extensive documentation provided on the CDC website describing the proper use of these weights (22). The survey package (23) can be used to perform these analyses in R. We provide a simple example in the next section, and more extensive examples are available in the vignettes for the nhanesA package.

## Results and Examples

We now demonstrate the use of the nhanesA R package through some examples. To download data from NHANES, we first need to know the relevant table names. NHANES table names typically consist of a base table name and a suffix; the suffixes _A, _B, _C, and so on generally correspond to NHANES cycle years from 1999–2000, 2001–2002, 2003–2004, etc. However, it is important to highlight that not every table strictly adheres to this naming convention. For instance, while DEMO_B and DEMO_C are associated with the 2001–2002 and 2003–2004 cycles, respectively, the corresponding table for the 1999–2000 cycle is named ‘DEMO’, without the _A suffix. While this pattern holds for most tables, certain tables such as SSAFB_A and SSOL_A from the 1999–2000 cycle do include the _A suffix. To assist users in navigating these variations, the nhanesA package includes the nhanesSearchTableNames() function, which allows users to easily locate all table names containing a specific string, optionally including details such as cycle and publication date, thus simplifying the process of identifying relevant table names.

**Figure:**
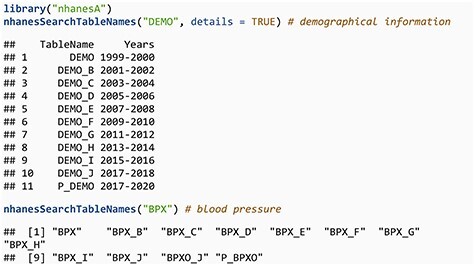


The second example shows a change in naming convention, related to a protocol change in how blood pressure is measured. Tables with a P_ prefix merit special mention. During the 2019–2020 cycle, data collection was disrupted by the COVID-19 pandemic. Therefore, the partial 2019–2020 data (herein 2019–March 2020 data) were combined with data from the previous cycle (2017–2018) to create a nationally representative sample covering 2017–March 2020. These data files have the same basic file name, e.g. DEMO, but add the prefix P_. These ‘pre-pandemic’ files require special handling, and the CDC has provided substantial guidance as well as updated survey weights.

### Downloading and combining data

We next look at average blood pressure for individuals over 40 years of age by reported ethnicity for the 2017–2018 cycle. For that, we first download and merge the demographic data (DEMO_J) and the blood pressure data (BPX_J) corresponding to this cycle.

**Figure:**
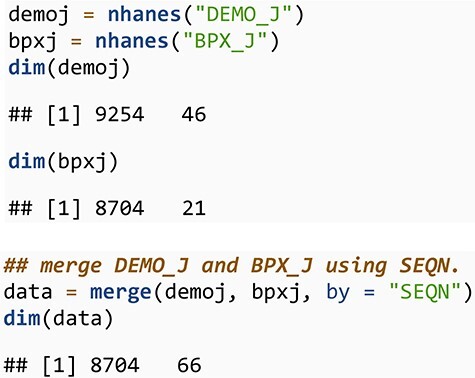


### Use of survey weights

In order to make appropriate estimates, we will need to create a survey design object to incorporate the weights into our analysis. It is essential to create the survey design structure prior to doing any subsetting of the data. This ensures that the complex survey design features, such as stratification and clustering, are accurately captured and applied to the entire dataset. In the code below, we use tools in the R package survey (23). We refer the reader to the documentation for that package for details and specific recommendations. The CDC provides detailed explanations on how to use survey weights in (22).

**Figure:**
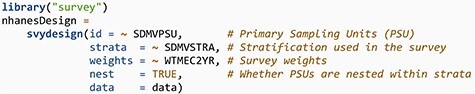


Next, we subset the data to contain only subjects over 40 years of age. We use the tools in the survey package so that appropriate adjustment of weights is made. We also create a second naive subset that ignores the survey design to easily examine the unadjusted values.

**Figure:**
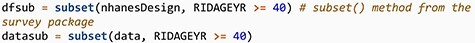


For illustration purposes, we examine diastolic blood pressure and for ease of presentation we only use the first measurement, variable BPXDI1 in table BPX_J. We can compute the unadjusted mean of diastolic blood pressure both for the whole table and also split by ethnicity.

**Figure:**
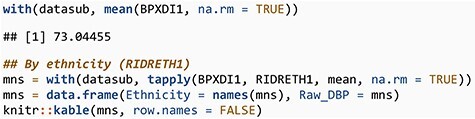


**Table:** 

Ethnicity	Raw_DBP
Mexican American	72.41000
Non-Hispanic Black	75.71466
Non-Hispanic White	70.84130
Other Hispanic	72.97611
Other race—including multiracial	74.41311

Next, we perform the same analysis using the survey weights. First, we compute the adjusted overall mean for the population represented by the data in the table and then also compute adjusted means for each ethnicity and present both those estimates and the unadjusted estimates computed above in a single table.

**Figure:**
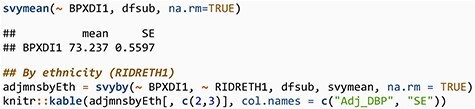


**Table:** 

	Adj_DBP	SE
Mexican American	74.03194	0.5572277
Non-Hispanic Black	75.71874	0.7416767
Non-Hispanic White	72.45422	0.6870412
Other Hispanic	74.73756	1.2303946
Other race—including multiracial	74.60215	0.5949430

**Figure:**
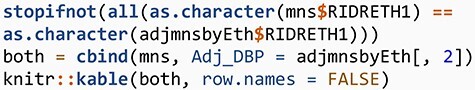


**Table:** 

Ethnicity	Raw_DBP	Adj_DBP
Mexican American	72.41000	74.03194
Non-Hispanic Black	75.71466	75.71874
Non-Hispanic White	70.84130	72.45422
Other Hispanic	72.97611	74.73756
Other race—including multiracial	74.41311	74.60215

### Challenging aspects of the NHANES data

There remain some challenges to analyzing the NHANES data for which there are no easy solutions. We discuss a number of the issues here in order to alert analysts to their existence so they can remediate any impacts.

**Figure 3.**
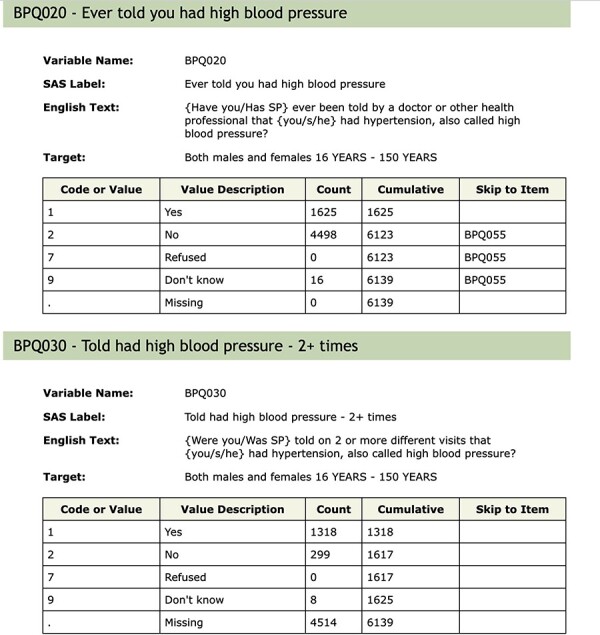
Questions BPQ_020 and BPQ_030 from table BPQ_D.

Within NHANES, there is a substantial amount of missing data. In part, this arises from non-response, but it can also arise due to the fact that not all respondents participate in all of the assays, exams or questionnaires. In other settings, missingness can be introduced by the process used to deliver the survey. We show an excerpt of the blood pressure and cholesterol documentation for 2005 to 2006 in Figure 3. We can see that anyone who answered either ‘No’ or ‘Don’t know’ to question BPQ_020 (Ever told you had high blood pressure) skipped over the question BPQ_030 (Told had high blood pressure - 2+ times), as it makes little sense for them. Importantly, the value stored in the database for those people for BPQ_030 was a missing value. Now, in some circumstances, an analyst might prefer to assume that if the respondent had not been told that they had high blood pressure once, they also had not been told they had high blood pressure two or more times. They would then fill in those missing values as ‘No’ so that they had more complete case information for their analysis. There are many instances in the NHANES data where questions are skipped as part of the survey delivery and it is important that the analyst tries to detect those and make reasonable assumptions for the analysis.

In the NHANES dataset, data coarsening is frequently observed. For instance, the age variable RIDAGEYR uses a representation where ages over 85 years are recorded as the value 85 in the first four cycles, and ages over 80 years are recorded as 80 in subsequent cycles. Similarly, the ratio of family income to poverty (INDFMPIR) uses the value 5 to indicate a ratio ≥5.00. These practices, adopted by the CDC keeping privacy issues in mind, nonetheless compromise the precision of numerical values in the dataset and require careful handling. Additionally, for variables such as BMI, the inclusion of children in the survey provides special challenges as their interpretation requires the use of age-specific reference values.

As noted earlier, the 2019–2020 cycle data collection was disrupted by the pandemic, and to create a nationally representative sample, the CDC combined the ‘partial’ data with data from the previous cycle. These datasets require special handling, and particular care should be taken before combining data from this release with data from other cycles.

## Other software tools to obtain NHANES data

### R packages

There are several other R packages that pertain to NHANES, including nhanesaccel (24), AsthmaNHANES (25), NHANES (26) and RNHANES (27). The first three pertain to specific subsets of NHANES data and are not designed for comprehensive access and use. The RNHANES package has functions to download and process continuous NHANES data; however, the package has not been updated to accommodate the pandemic-affected data.

The NHANES package (26) provides a subset of data from the 2009–2010 and 2011–2012 cycles. The authors have created a small subset of the data for teaching purposes. They have included 75 variables and created two datasets. The NHANESraw dataframe is the raw data together with information on the sample-weighting scheme. Their NHANES dataframe contains 10 000 rows that were resampled from NHANESraw that ‘accounted for’ the oversampling, and hence, analyses using NHANES can be performed without using the survey weights. The authors are quite explicit that this is a teaching resource and that any scientific investigations should rely on the data from the NHANES CDC site and not on their subset.

The RNHANES package (27) is produced by the Silent Spring Institute. RNHANES provides an easy way to download and analyze data from NHANES with a focus on the laboratory data. They provide methods to find all data files and to download them. They provide a search capability as well as making some attempt to obtain the units of measurement for the laboratory data. The nhanes_load_data() function provides a method for downloading and merging data, although the features are limited. It also has arguments to allow for recording/translating factor variables although that seemed to be very slow to run. There are useful functions that encapsulate the use of the survey package but that seems to be at the expense of flexibility in the analysis.

### Stata

We did not find any Stata modules or packages, but there are good resources available on the web, such as those from the Statistical Consulting Unit at UCLA (28).

### Python

We are aware of two actively maintained Python libraries for working with NHANES data: nhanes-dl (29) and pynhanes (30). In Python, one can use Jupyter notebooks to achieve reproducible results. Jupyter notebooks, similar to Rmarkdown, allow for an organized presentation of text, code and their respective outputs (including plots) within a single document. This facilitates reproducibility, enabling readers to easily replicate and understand the presented work. The nhanes-dl library is designed to download continuous NHANES codebooks and convert them into ready-to-use pandas dataframes, although its documentation is somewhat lacking. The pynhanes package offers several Jupyter notebooks on its GitHub repository (31) to demonstrate its usage.

## Discussion and future work

NHANES, with its depth and breadth of health and nutritional data, serves as a cornerstone for ‘epidemiological’ and health research. However, the intricacies and nuances associated with the data, combined with the varied methodologies employed across different research domains, present considerable analytic challenges. We have described a number of ways in which the nhanesA R package can facilitate analyzing these data and have indicated a number of issues that are not easily addressed in software and remain for the analyst to address.

We believe that there is an additional value to be obtained from the many papers based on NHANES and, in particular, point out that when the reported analyses are reproducible then they also become extensible in at least two directions. First, when studying population characteristics, there is substantial value in being able to repeat an analysis when data from a new cycle are released. Second, for any analysis, the ability to extend that analysis using additional covariates from other questionnaires or to explore the impact of ‘not’ adjusted for covariates (e.g. explore social determinants of health) can be very powerful.

With regard to reproducibility, we mean the computational reproducibility of the figures and tables in a paper, which essentially means that once the dataset is agreed upon, all analytical outputs can be precisely replicated, while the general scientific reproducibility emphasizes the need to obtain similar results across analogous, though not identical, samples. In the supplement, we propose a process that offers a structured approach for researchers using the NHANES dataset. Harnessing the synergy between GitHub, Rmarkdown/Quarto, and specific packages like nhanesA, we set the stage for a transparent, modular, and rigorously organized research process. Every stage, from data selection to preprocessing decisions and analytical procedures, is systematically recorded and versioned, ensuring transparency and reproducibility. The essential components of this process have been used to write papers and books by many of the contributors to the Bioconductor Project (32) for the past 20 years or so. We believe that it would be valuable to start a community effort to collect and collate papers based primarily on NHANES data that use strategies to encourage reproducibility and extensibility, regardless of the computing language used.

We believe that encapsulating the public NHANES data into a SQL database that is contained in a Docker (33) container is an important next step. This would enable faster access, both due to the data being local to the user and also because the use of SQL and various tools that come with databases better support some of the data manipulations. We are working on a container that also includes an instance of R and RStudio (34) to further encourage reproducibility of results. Such an approach will make it easier to add data resources and create more complex and hence more valuable data sets.

## Figures and Tables

**Figure 4.**
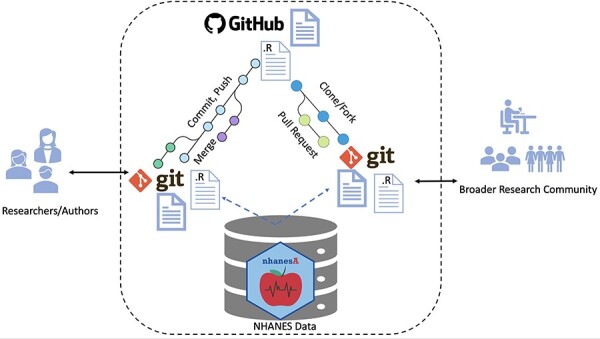
Workflow for ensuring transparent and reproducible research: (i) authors use Rmarkdown and R files, managed with Git version control for organization and collaboration. The nhanesA package facilitates NHANES access. Git and GitHub facilitate this by archiving and source code control. (ii) Work is committed, pushed and made public on GitHub in the form of Rmarkdown and R files. (iii) Anyone who wants to reproduce the work can fork or clone the repository to reproduce or expand upon the work. External users can access the NHANES database in the same way as the original authors. Contributions or extensions can be integrated via pull requests and subsequent merging.

## Data Availability

The nhanesA R package is available to the public on: https://github.com/cjendres1/nhanes. The current CRAN version is also available at https://cran.r-project.org/package=nhanesA.

## Appendix

### An example approach to reproducible research with the nhanesA R package

We believe that the nhanesA R package makes a substantial contribution to enhancing reproducibility and rigor in the scientific process. Here, we want to outline a few tools that can be used in conjunction with the nhanesA ‘R package’ to create documents that are reusable and extensible. The reproducibility of a paper, or result, can be enhanced by using a number of tools and processes that are commonly used for software development. We describe a set of tools, which we believe are useful and then outline a simplified paper-writing approach that uses these tools.

An important development was the concept of ‘Markdown’ (35), which is a straightforward markup language designed for crafting formatted text without the intricacies of HTML. Rmarkdown, and more recently Quarto, builds upon Markdown, intertwining it with the R programming language. Essentially, Rmarkdown is an implementation of Markdown, allowing users to embed code within a document. This fusion supports dynamic reporting, where narrative and code coexist, fostering clear, reproducible research outcomes.

Xie *et al*. (14) and Allaire *et al*. (36) describe systems that integrate software (code) and text. These can be thought of as explicit descriptions of how the figures and the tables in the published paper were created. Rmarkdown documents are processed by different ‘engines’ that transform them into specific outputs such as a PDF file for publication or HTML output for putting on the web.

A second important tool to help with reproducibility is the use of version control systems. These were originally developed for software development, but they work equally well for writing papers. A widely used tool for version control is GitHub (37). As shown in Figure 4, one example of using this approach based on R (38) is ‘The Epidemiologist R Handbook’ (39), which is written in Bookdown (15) and is maintained in GitHub (40). The authors have created an entire textbook using Markdown, and they use GitHub to handle version issues as well as bug reporting and fixing. This approach has been used widely in the R community for over 20 years with substantial success. For example, the authors of Tidyverse (41) manage a set of popular data science tools with GitHub. It should come as no surprise that this paper is also written in Markdown and uses GitHub as its source code repository (42). Depending on their preferences, researchers can also use alternative authoring workflows such as those based on Sweave (13) or Quarto (43) and alternative source code management systems such as Subversion (44).

### Sample design of NHANES

Zipf *et al*. (3) provide the following description of the NHANES sampling design. Anyone analyzing NHANES data should work with sample survey experts to ensure that their models are accurately accounting for the sampling design.

NHANES was designed to assess the health and nutritional status of the civilian noninstitutionalized. NHANES data were not obtained using a simple random sample. Rather, a complex, multistage probability sampling design was used to select a sample representative of the civilian noninstitutionalized household population of the USA. Sample selection for NHANES followed these stages, in order: (i) selection of PSUs, which are counties or small groups of contiguous counties. (ii) Selection of segments within PSUs that constitute a block or group of blocks containing a cluster of households. (iii) Selection of specific households within segments. (iv) Selection of individuals within a household, see the ‘Household Interview’ section for more information on sample person selection.

### A simplified NHANES paper-writing process

Here, we sketch out an outline for writing a paper using the tools we mention in order to create a reproducible paper. By reproducible, we really mean that once we have agreed on the data to analyze that all of the tables, graphs and other analyses can be reproduced exactly. Now, this is not the concept of scientific reproducibility where one expects to find a similar result when the basic experiment is repeated, under similar but not identical circumstances, but it is an important goal in and of itself.

One would first create a new GitHub repository for the project. Then, identify the variables of interest and the questionnaire files they are in as well as the cycles (years) of data that will be used. Create an Rmarkdown document and in that use the nhanesA package to download the relevant data. The author will then check that document into the GitHub repository so that all updates and modifications are noted and so that collaborators can check out the document.

At this point, you will start to write code chunks in the document to first transform and filter the data according to the entry criteria for your study. For example, you might want to look at blood pressure on adults over 40 years. On examining the Blood Pressure (BPX) tables you find that two different blood pressure measurements (systolic and diastolic) were recorded at two different time points. You have to decide how to process those data. Do you take only one, or do you average both? What about people who have only one measurement? Do you keep them or remove them? All of these decisions will impact the analysis and the actual values you report in your paper. By including the code to do this processing in your Markdown document and reader can check the code for the actual steps you took.

Then, as your research progresses, you will manipulate the data to compute different summary statistics, perhaps mean diastolic blood pressure by reported ethnicity. Again, the specific details of how you did that will be maintained in the Markdown document. Ultimately, you will have finished your analysis and then arrange the outputs, using the tools available for processing Rmarkdown to produce the final paper for publication, which you can then submit. And make sure you commit everything you need (images, tables, text, etc.) to your GitHub repository.

Once the reviews come back, you will update and modify that code and text to reflect the changes that have been asked for. And again, you will check in all the files and changes. Once your paper is published, you can refer interested parties to your GitHub repository where they can download the Markdown documents and rerun them. Perhaps, they will make changes to your assumptions to see whether the results change.

These tools, though demanding an initial learning curve, are intuitive and efficient. As more researchers embrace these practices, the collective reliability and robustness of NHANES-based research will undoubtedly be enhanced. By fostering an ecosystem of transparent, replicable and collaborative research, we can reach more informed decisions, richer insights and a deeper understanding of the NHANES.
